# Antidiabetic Drugs in the Treatment of Alzheimer’s Disease

**DOI:** 10.3390/ijms23094641

**Published:** 2022-04-22

**Authors:** Michalis Michailidis, Despina A. Tata, Despina Moraitou, Dimitrios Kavvadas, Sofia Karachrysafi, Theodora Papamitsou, Patroklos Vareltzis, Vasileios Papaliagkas

**Affiliations:** 1 Laboratory of Psychology, School of Psychology, Aristotle University of Thessaloniki, 54124 Thessaloniki, Greece; michalismichailidis93@gmail.com; 2 Laboratory of Cognitive Neuroscience, School of Psychology, Aristotle University of Thessaloniki, 54124 Thessaloniki, Greece; dtata@psy.auth.gr (D.A.T.); despinamorait@gmail.com (D.M.); 3 Histology and Embryology Department, Faculty of Medicine, Aristotle University of Thessaloniki, 54124 Thessaloniki, Greece; kavvadas@auth.gr (D.K.); sofia_karachrysafi@outlook.com (S.K.); thpapami@auth.gr (T.P.); 4 Department of Chemical Engineering, Aristotle University of Thessaloniki, 54124 Thessaloniki, Greece; pkvareltzis@cheng.auth.gr; 5 Department of Biomedical Sciences, School of Health Sciences, International Hellenic University, 57400 Thessaloniki, Greece

**Keywords:** amyloid beta, Alzheimer type 3 diabetes mellitus, intranasal insulin, metformin, type 2 diabetes mellitus, incretins, PPARγ agonists, thiazolidinediones

## Abstract

The public health burden of type 2 diabetes mellitus and Alzheimer’s disease is steadily increasing worldwide, especially in the population of older adults. Epidemiological and clinical studies suggest a possible shared pathophysiology between the two diseases and an increased risk of AD in patients with type 2 diabetes mellitus. Therefore, in recent years, there has been a substantial interest in identifying the mechanisms of action of antidiabetic drugs and their potential use in Alzheimer’s disease. Human studies in patients with mild cognitive impairment and Alzheimer’s disease have shown that administration of some antidiabetic medications, such as intranasal insulin, metformin, incretins, and thiazolidinediones, can improve cognition and memory. This review aims to examine the latest evidence on antidiabetic medications as a potential candidate for the treatment of Alzheimer’s disease.

## 1. Introduction

Alzheimer’s disease (AD) is a chronic neurodegenerative disease that is the most common type of dementia and is mainly characterized by decline in cognitive ability and impaired memory, as well as changes in personality and behavior [1]. According to recent reports, 5.8 million Americans aged 65 and older have AD today, a number projected to rise to 13.8 million by mid-century in the USA alone [2]. The strongest genetic risk factor for AD is APOE4 and the pathological characteristics are β amyloids [3,4]. It has been estimated that 25% of the population are carriers of APOE4 [5].

Diabetes mellitus (DM) is one of the most prevalent chronic metabolic conditions, with devastating complications and increased risk of premature death. In 2019, approximately 463 million individuals were affected by DM [6]. The most prevalent subtype of diabetes is type 2 diabetes mellitus (T2DM), which is mainly characterized by high blood glucose levels (hyperglycemia) and insulin resistance [7].

T2DM has also been associated with an increased risk of dementia [8,9], in particular, AD by 45–90% [10,11]. The Rotterdam study was among the first to show an elevated risk of dementia with T2DM [12]. Moreover, it has been shown that patients with T2DM have a higher risk of amnestic mild cognitive impairment (aMCI) [13]. People who experience cognitive impairment combined with AD, when compared to people who experience only cognitive impairment, appear to be affected by the DM type and related complications as well as the antidiabetic treatment they receive [14]. Insulin resistance and hyperglycemia as features of T2DM have a detrimental effect on cognitive abilities [15], since insulin and insulin-like growth factor, also called somatomedin C (IGF-1), play an important role in cognitive ability, neural function, and development [16].

Recent research shows that AD shares many common links with diseases related to insulin resistance, such as neuroinflammation, insulin signaling disorder, oxidative stress, advanced glycosylation end products (AGEs), mitochondrial dysfunction, and metabolic syndrome [17]. Therefore, AD could be considered a metabolic disease caused by insulin and IGF-1 resistance in the brain, so the term type 3 DM was proposed [18]. Type 3 diabetes is, in essence, the failure of brain cells to respond to insulin, resulting in impairments in synaptic function, metabolism, and the immune response. The interaction between insulin signaling and AD or cognitive impairment can be also demonstrated by research data showing improvements in the cognitive function of AD patients after the administration of antidiabetic drugs such as intranasal insulin, metformin, thiazolidinediones, and incretins. Based on the studies that support the concept that AD is a metabolic disease of the brain [19,20,21] and the emerging evidence of a common pathophysiology between AD and T2DM, there has been a great interest in exploring whether antidiabetic medications currently approved for T2DM could be beneficial for AD treatment [22].

Numerous clinical studies have examined the extent of the effect that antidiabetic drugs have on the pathological manifestations of AD [23,24,25,26], while animal studies have shown beneficial effects on tau protein pathology [27,28] and β-amyloid [29,30], in neurogenesis [31], oxidative stress [32], synaptic function [33], cognitive function [34,35,36], and in neuroinflammation [37]. Findings of the greatest clinical interest have originated from clinical trials in patients with AD or MCI, which explored the hypothesis that antidiabetic drugs may be a neuroprotective treatment approach against AD. The aim of this review is to assess the efficacy of antidiabetic drugs in AD treatment.

## 2. Methods

### 2.1. Literature Search

The PubMed/MEDLINE and Google Scholar electronic databases were searched using the keywords “amyloid beta”, “Alzheimer type-3-diabetes”, “intranasal insulin”, “metformin”, “type 2 diabetes mellitus”, “incretins” and “PPARγ agonists”. A systematic search of the literature published between 2005 and 2020 was conducted, and two independent reviewers evaluated the studies. The database search lasted from November 2019 to February 2020. 

### 2.2. Inclusion Criteria

The articles that were included in this review fulfil the following criteria: (a) The subjects received treatment for AD or/and a T2DM treatment, if the expected outcome concerned risk of cognitive decline or dementia. (b) Age of study participants > 50 years old. (c) The type of studies included in this review were randomized clinical trials, population-based observational or case–control studies, prospective cohort studies, as well as reviews and meta-analyses. (d) Articles included were written in English.

### 2.3. Study Selection Chart

**Figure 1:**
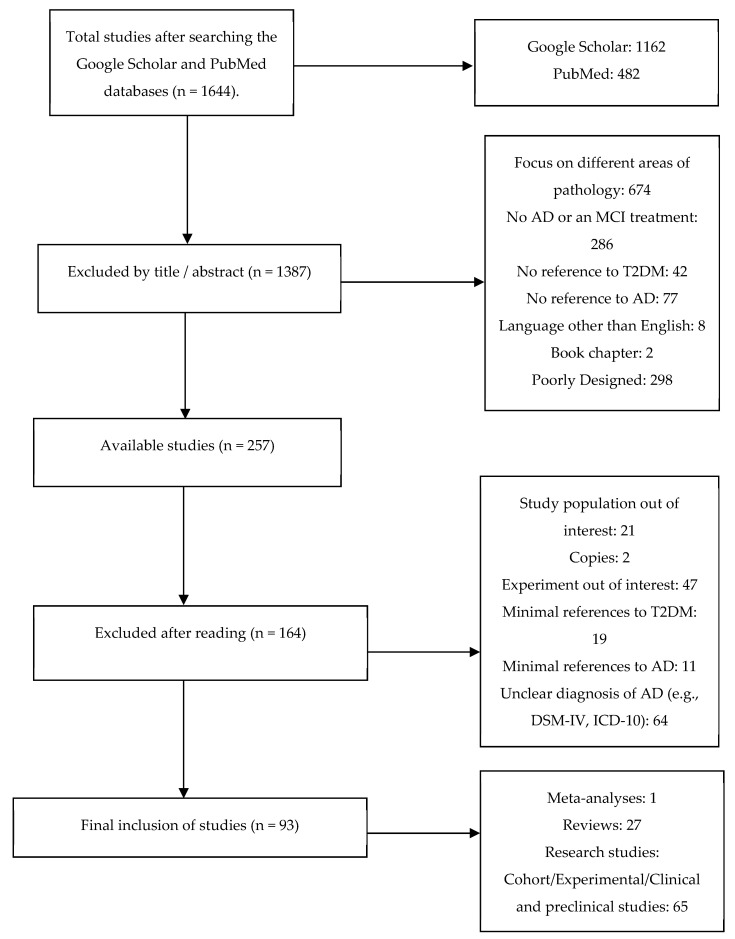
PRISMA flowchart of study selection.

Included and excluded studies were collected following the Preferred Reporting Items for Systematic Reviews and Meta-Analyses (PRISMA) flow [38] and depicted in Figure 1 below.

## 3. Results and Discussion

The main characteristics of the included studies are presented in Table 1

### 3.1. Intranasal Insulin

**Figure 2:**
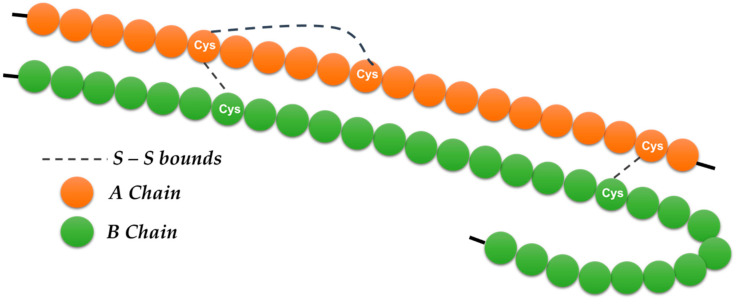
Insulin is a polypeptide hormone, relatively “small”, consisting of two peptide chains (A and B) containing a total of 51 amino acids, 21 amino acids in the A chain and 30 amino acids in the B chain. Of the 20 amino acids, it lacks the amino acids tryptophan (Try) and methionine (Met). It has three disulfide bridges (-S-S-), of which two hold the two chains. Neither of the two chains separately exhibits any physiological activity, and therefore the action of insulin is due to the overall configuration of its molecule (tertiary structure) and not to its individual components’ peptides or amino acids.

Insulin performs many important functions in the brain (Figure 2) related to food intake regulation, body weight, eating habits, and energy homeostasis [53,54].

It was proposed that AD might be a metabolic disease of the brain, driven by insulin resistance and insulin-like growth factor (IGF-1) resistance [19,20].

**Figure 3:**
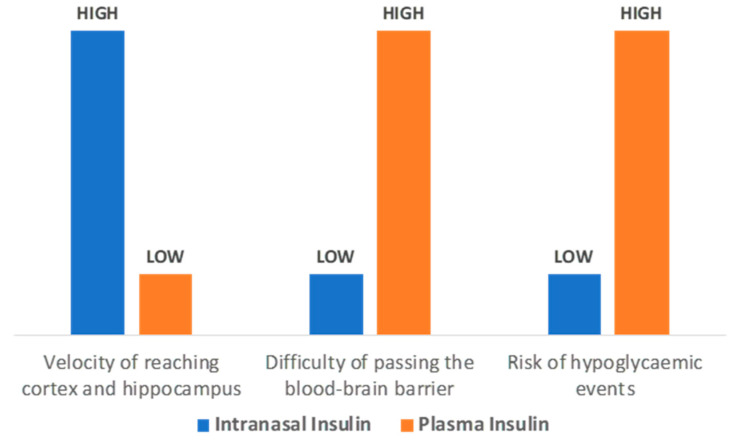
Intranasal insulin administration benefits for testing the cognitive improvement on AD and MCI patients.

Several studies have shown that insulin administration in AD patients reduces the action of kinases that promote tau protein hyperphosphorylation and enhances β-amyloid clearance and synaptic plasticity [55,56]. In fact, an earlier study by Craft et al. [57] showed that in the case of elevated insulin without hyperglycemia, memory was enhanced in AD patients, thus supporting the important role of insulin in memory improvement. Consequently, it has been hypothesized that increasing insulin function in the brain might counterbalance AD pathology. However, peripheral administration of insulin in order to reach the brain carries the risk of hypoglycemic events and the difficulty of passing the blood–brain barrier. On the other hand, intranasal insulin avoids the risk of hypoglycemia as it bypasses the blood–brain barrier [58] and through the nasal passages reaches the cortex and hippocampus within 15–30 min (Figure 3) [59].

**Figure 4:**
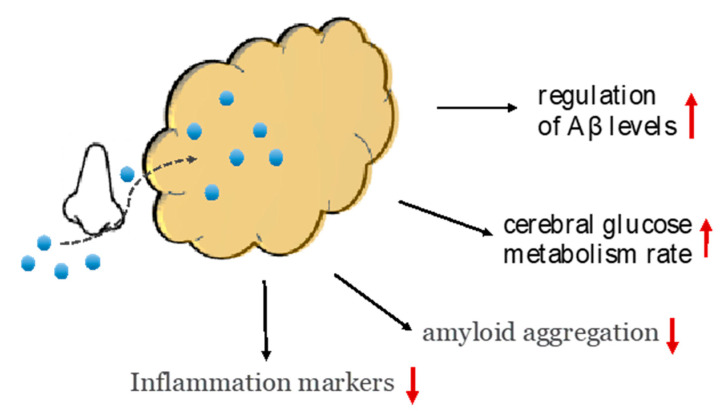
Intranasal insulin administration therapeutic evidence is based on several mechanism such as the reduction in β amyloid and general inflammation marker. Intranasal insulin bypasses the blood–brain barrier, which leads to the regulation of Aβ levels and cerebral glucose metabolism rate.

In a small (n = 24) pilot study [25] that examined a 3-week intervention in patients with MCI or early AD and compared intranasal insulin with placebo, improvements in working memory and cognitive skills were found due to intranasal insulin. Moreover, in a study by Craft et al. [39] chronic administration of intranasal insulin for 4 months in 104 patients with MCI or mild to moderate AD improved cognitive and functional ability, with these changes being associated with alterations in levels of β-amyloid but also in the CSF β-amyloid/tau protein ratio. Insulin has been shown to inhibit the deterioration of the cerebral glucose metabolism rate in specific areas of the brain [39]. It should be noted that in this study, intranasal insulin appeared to be an effective therapeutic approach for patients with AD, with no side effects due to prolonged treatment (Figure 4) [39].

Some of the clinical trials evaluated fast-acting forms of insulin while others tested longer-acting insulin analogs. In a more recent study [60] in which researchers compared NPH insulin to insulin detemir and placebo in adults with MCI or AD, NPH insulin appeared to improve memory after 2 and 4 months compared to the placebo, while no significant effects of long-acting insulin were observed compared to the placebo. In addition, NPH insulin administration was associated with a decrease in tau-P181/β-amyloid ratio; however, various genetic factors such as APOE4 status affected insulin levels and insulin resistance [60].

APOE4 is the strongest genetic risk factor for AD [3], and about 25% of the population carries at least one ε4 allele [5]. There has been an improvement following insulin administration in the cognitive function of AD patients who were not ApoE4 carriers, while no improvement was found in patients with APOE4; in some cases, the symptoms of the disease worsened [25,26]. A recent study by Claxton et al. [24] examined responses to intranasal administration of insulin detemir to MCI and AD patients, who showed improvements in cognitive, verbal, and audiovisual memory. In this study, APOE4 played an important role in the results, and, in contrast to the aforementioned study [25], it seemed that the responses were regulated differently. Significant improvements in verbal memory and peripheral insulin resistance levels in APOE4 carriers were observed after three weeks of treatment, while no improvements were observed in ApoE4 non-carriers [24].

In an ongoing Phase II/III clinical trial with the acronym SNIFF (Study of Nasal Insulin in the Fight Against Forgetfulness) [61], two different insulin delivery devices were used in order to deliver 20 IU of insulin or placebo after breakfast and dinner to 240 patients with either MCI or early AD. After one year of treatment, no statistically significant effect of intranasal insulin on cognitive abilities was found in the main cohort of 240 patients who used one of the two devices. Nonetheless, a group of 49 patients who used another device exhibited a slowing of worsening in the subscale of ADAS-COG-12 and daily life activities at one year [61]. It should be noted that in this study, the change in the insulin delivery device in the middle of the experiment may have played an important role and may have affected the results.

### 3.2. Metformin

**Figure 5:**
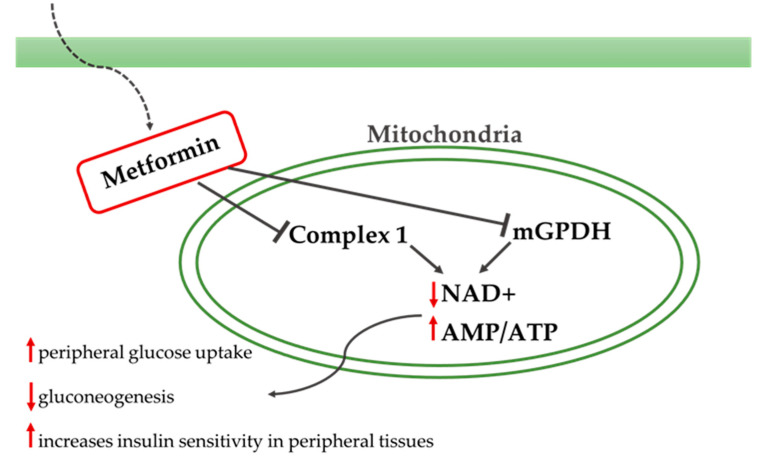
Metformin’s mechanism and signaling. Metformin acts in the liver, reducing hepatic glucose production by inhibiting gluconeogenesis and glycogenolysis. Metformin also acts in the muscles by increasing insulin sensitivity and improving peripheral glucose uptake and delays the absorption of glucose from the intestines. Metformin inhibits the mitochondrial respiratory-chain complex 1 and the mitochondrial glycerol phosphate dehydrogenase (mGPDH) leading to a reduction in NAD+ and ATP and to the above-described results.

**Figure 6:**
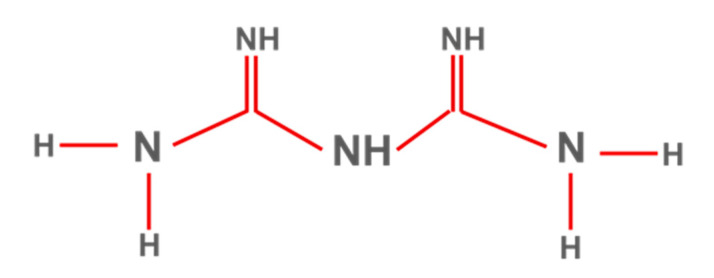
Metformin’s chemical structure.

Metformin is a biguanide that increases peripheral glucose uptake, suppresses gluconeogenesis in the liver, and increases insulin sensitivity in peripheral tissues (Figure 5). Metformin is the first drug prescribed in patients with T2DM, mainly due to the beneficial effects observed on hemoglobin A1c levels, weight, and cardiovascular mortality, as well as due to its safe action profile (Figure 6) [62]. Currently, clinical research data on the use of metformin in AD are limited, and the results are inconclusive.

Currently, clinical research data on the use of metformin in AD are limited and the results are inconclusive. Several studies in the last decade have shown that metformin may significantly improve cognitive dysfunction in patients with T2DM [63,64]. Moore et al. (2013) [44] observed an increased risk of cognitive impairment in patients with T2DM after long-term metformin treatment. On the contrary, Ng et al. [40] reported that metformin reduced the risk of cognitive impairment in T2DM patients, aged 55 years and older, who were monitored for more than 4 years. In the first study [44], it is possible that the negative results were due to vitamin B12 deficiency. According to the authors of this study [44], vitamin B12 and calcium supplements alleviated the aforementioned vitamin B12 deficiency and had beneficial effect on cognitive function. In a study from Taiwan’s National Health Insurance that contains a large database of structured data about people aged 50 years and over, some of whom (n = 25,393) were diagnosed with T2DM and others were undiagnosed (n = 101,816), it was found that dementia prevalence increased by 2.6 times in patients with T2DM [41]. In particular, it was found that metformin reduced dementia risk by 24% compared to patients who had not used any antidiabetic medication. In a small randomized control trial, a significant positive effect of metformin on executive function was found as well as some improvements in memory and attention, while there was no effect of metformin on CSF AD biomarkers [42]. In contrast to the above evidence, in a case–control study of diabetic individuals (n = 7086), which assessed the risk of AD in relation to the type of antidiabetic drugs, it was found that long-term use of metformin caused a slight increase in AD risk, while no such effect was observed following long-term use of sulfonylureas, thiazolidinedione, or insulin [45]. A possible explanation for this increased risk of AD and cognitive impairment may be a vitamin B12 deficiency, often seen after metformin use.

Based on the above evidence, there is high need to further investigate the role of vitamin B12 deficiency. Another important issue is the route of administration, since drugs such as metformin have been administered only via systemic routes and, as a consequence, their action depends on their ability to cross the blood–brain barrier (but also from the peripheral insulin levels). Given the widespread use of metformin and its effect on cognitive functions, additional research is needed, in particular, a long-term study with adequate sample or a meta-analysis of smaller studies in order to further elucidate its action.

### 3.3. Incretins

**Figure 7:**
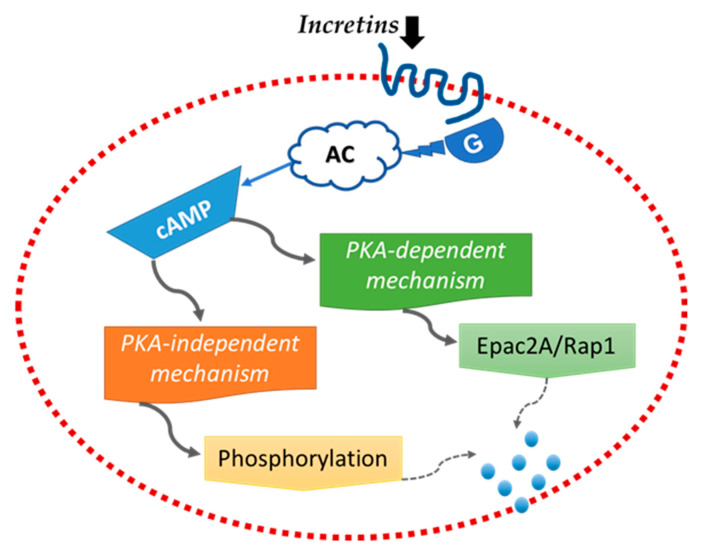
Incretins’ mechanism and signaling in the b-cells of pancreas. Incretins induce the cAMP signaling pathway through G protein-coupled receptors. The cAMP signaling is divided into two different Protein Kinase A pathways: the dependent mechanism activates the exocytosis of insulin, while the independent regulates the amount of insulin granules that prepared for exocytosis.

Incretins, including glucagon-1 peptide (GLP-1) and glucose-dependent insulin-releasing polypeptide (GIP), are important metabolic hormones responsible for the expression of the insulin gene, proliferation of ng β-cells, and lowering glucose levels by stimulating insulin secretion mechanisms (Figure 7) [65].

GLP-1 is secreted by the gut in response to food intake, and its receptors (GLP-1Rs), expressed in pancreatic β-cells, enhance insulin release in response to high glucose levels. Following the secretion of the GLP-1, the enzyme dipeptidyl-peptidase 4 (DPP4) degrades the GLP-1 within minutes. Therefore, GLP-1 analogs, which are resistant to the enzyme DPP4, have been developed for clinical use, and GLP1-R receptor agonists (liraglutide, exentin-4) have been approved for use in patients with DM [66]. GLP-1 and its receptors are not found exclusively in the pancreas and vascular endothelium but are also expressed in the brain and specifically in the hippocampus, hypothalamus, cerebral cortex, and olfactory bulbs [67]. The role of incretins and incretin analogues in the brain is neuroprotective [68], as they enhance cell proliferation, memory, and synaptic plasticity, while reducing β-amyloid plaques, oxidative stress, and inflammation [69,70,71].

**Figure 8:**
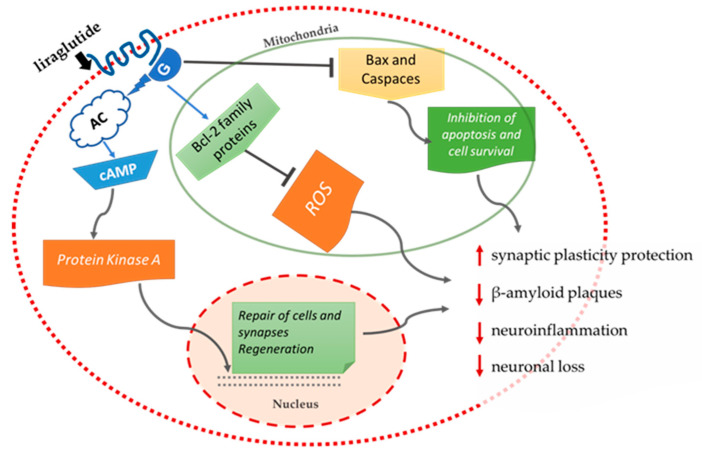
Liraglutide’s preventive and therapeutic properties of AD based on evidence of several studies [70,71,72,73,74].

Long-acting liraglutide has been shown to normalize the distribution of cell membrane insulin receptors in a rat model with AD (APPSWE/PS1dE9), thus improving insulin signaling disorders [72]. In addition, systematic administration of liraglutide in transgenic mice with AD for 8 weeks prevented the underlying neurodegenerative effects observed in AD, such as neuronal loss, memory impairment, and a decrease in synaptic plasticity in the hippocampal region [73]. In particular, liraglutide reduced the deposition of β-amyloid plaques by 40–50%, while a decrease was also observed in the inflammatory response based on activated glial cells [73]. In mice that received intrahippocampal injections of β-amyloid, it was observed that pretreatment with liraglutide before injection was a protective factor against impairments in spatial memory and long-term potentiation (LTP) induced by β-amyloid [74]. Additional experiments in transgenic mice have shown that liraglutide promotes neurogenesis, has a positive effect on the cerebral microvascular system, and also reduces tau protein hyperphosphorylation in AD [75,76,77,78]. It also appears that liraglutide has not only preventive properties but also the ability to reverse several of the key pathological features that appear in the final phase of AD in mice models [79]. Positive results have also been observed in the rat model APPswe/PS1ΔE9 with AD, where the long-term administration of the analogue hormone liraglutide GIP (D-Ala2GIP) protects synaptic plasticity and memory formation and reduces β-amyloid plaques and neuroinflammation, while normalizing stem cell proliferation (Figure 8) [70].

Dipeptidyl-peptidase 4 (DPP4) enzyme inhibitors are also used as an alternative treatment. They can extend the action time of GLP-1 and GIP, thus regulating glucose in T2DM [80]. A study by Kornelius et al. [81], found that linagliptin (a DPP4 inhibitor) can restore the impaired insulin signaling induced by β-amyloid in neuronal cells, indicating the important therapeutic role that DPP4 inhibitors may play in the neurotoxicity of AD. Two other DPP4 inhibitors, saxagliptin and vildagliptin, showed similar efficacy when given orally to AD transgenic mice, resulting in reduced β-amyloid deposition, improved memory, and increased levels of hippocampal GLP-1, as well as reduced tau protein phosphorylation and markers of inflammation [82,83]. An alternative substance is exentin-4, a long-acting incretin GLP-1 receptor agonist, which has a neuroprotective effect in neurodegenerative diseases such as AD and Parkinson’s disease and is fully approved for use in patients with T2DM [32,69]. In an in vitro study by Bomfim et al. [35], the property of β-amyloid oligomers to attenuate axial transport was inhibited by the administration of exentin-4 (GLP-1R agonist), which appeared to improve cognitive ability by reducing the serine phosphorylation of the insulin receptor substrate (IRS-1) in the hippocampus. The only human study of liraglutide in AD patients [46], showed that a 6-month treatment had moderate neuroprotective effects, mainly expressed by improvements in cerebral glucose metabolism. In the same study, liraglutide administration had no effect on the β-amyloid deposition of AD patients when compared to placebo patients. 

Additional research is needed to clarify the role of incretins in the treatment of AD in humans. Despite promising evidence from animal experiments, existing studies have failed to demonstrate reversal of AD pathology in humans. More studies are necessary to determine the exact action of incretins at each individual stage of AD, in order to define the therapeutic window for these drugs.

### 3.4. Thiazolidinediones (PPARγ Agonists)

**Figure 9:**
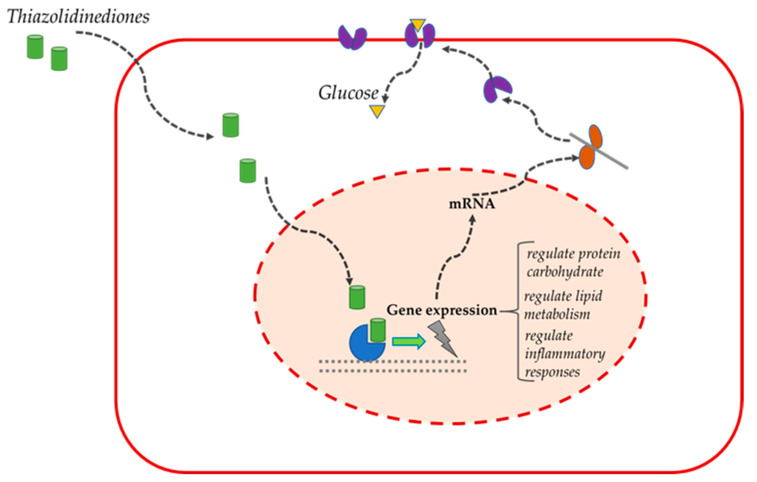
Thiazolidinediones’ basic mechanisms and signaling.

In patients with T2DM, PPARγ agonists reduce hyperglycemia, improve insulin resistance and cholesterol levels (Figure 9) [84].

**Figure 10:**
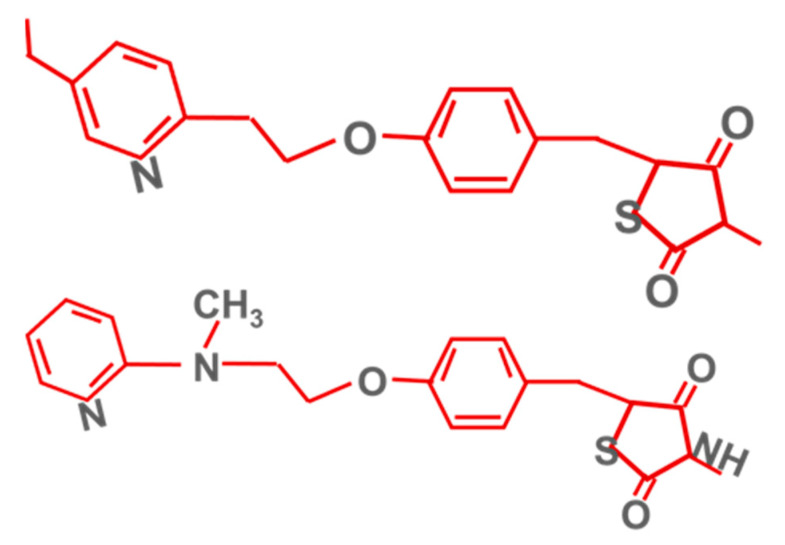
Pioglitazone’s (above) and rosiglitazone’s (below) chemical structures.

The best known PPARγ agonists are pioglitazone and rosiglitazone (Figure 10). 

The rationale for their use in AD patients is based on the increased expression of PPARγ in the temporal cortex of these patients compared to the control group [85]. PPARs have the ability, as nuclear hormone receptors, to regulate protein carbohydrate and lipid metabolism, as well as inflammatory responses [86], making their agonists a potential treatment for T2DM and insulin resistance in the brain [87], while the latest research suggests that PPARγ agonists have the potential to activate pathways in the brain that are regulated by IGF-1 [87].

A small pilot study of rosiglitazone in MCI and AD patients showed that treatment with rosiglitazone for 6 months resulted in improved attention and delayed recall compared to patients receiving a placebo [48]. In a larger study conducted shortly after by Risner et al. [49], in which different doses of rosiglitazone (2, 4, or 8 mg) were administered to patients with mild to moderate AD, a significant improvement in ADAS-Cog was observed following administration of 8 mg rosiglitazone to APOE4-negative patients only. In fact, APOE4-positive patients not only showed no improvement, but also, interestingly, they exhibited a cognitive decline in lower doses of rosiglitazone [49]. The exact way in which the APOE4 gene mediates the action of PPARγ agonists has not been adequately explored. In another study, responses to treatment with metformin, rosiglitazone, or a combination of the two were evaluated to determine if an improvement in insulin resistance could explain fluctuations in cognitive performance for 36 weeks in the elderly with MCI and T2DM [50]. The results showed that rosiglitazone in diabetic patients was more effective than metformin in protecting against cognitive impairment [50]. In addition, a pilot study by Sato et al. [52] on pioglitazone in patients with AD and T2DM found that administration of 15–30 mg pioglitazone for 6 months improved cognitive capacity and cerebral blood flow in the parietal lobe, compared with the control group. In the same study, pioglitazone administration was shown to reduce fasting plasma insulin levels, indicating increased insulin sensitivity [52]. Additionally, Hanyu et al. [51] found, despite the small sample size, that pioglitazone resulted in improvements in ADAS-Cog and metabolic function in patients with AD and TD2M. Another pilot study of the same year evaluated the safety of administration of pioglitazone over an 18-month period in patients with AD but without T2DM. Although treatment with pioglitazone was tolerable, the findings did not support its efficacy in these patients [88].

Finally, in relation to clinical findings in humans, a recent meta-analysis of PPARγ agonists in AD that included a total of nine studies showed that only pioglitazone could provide improvement in the early stages of AD and also in stages of mild to moderate AD [89]. In animal models, PPARγ agonists appear to improve various aspects of AD pathology including reduced β-amyloid expression, decreased expression of inflammatory genes [90], and neuroprotective activity related to calcium homeostasis in cultured hippocampal neurons [91].

Respectively, pioglitazone in animal models with AD had a beneficial effect. More specifically, it reduced cerebellar dysfunction [92], rescued synaptic transmission deficits, enhanced long-term memory [89], and restored dendritic density and neuroplasticity [93]. There have been studies with negative results as well, such as that of Gold et al. (2010) [47], in which no benefit was observed following the administration of rosiglitazone in patients with mild to moderate AD. Moreover, thiazolidinediones modulate Wnt signaling that is involved in Aβ-induced neurodegeneration in AD patients [94].

In summary, despite the proven benefits of thiazolidinediones in AD treatment, there were significant side effects mainly related to rosiglitazone, which consisted of edema, myocardial infarction, and stroke [95]. In 2010, due to these side effects, the USA and Europe restricted rosiglitazone use for T2DM treatment [94]. The above complications, as well as the lack of a large number of clinical trials, must be considered in order to ensure the application of thiazolidinediones in future treatments.

### 3.5. Discussion

In the present study, we investigated the use of antidiabetic drugs for AD prevention and treatment. Drugs such as metformin, intranasal insulin, thiazolidinediones, and incretins have shown some beneficial effects both on humans and mice. The latest research suggests that thiazolidinediones have the potential to activate pathways in the brain that are regulated by IGF-1; however, rosiglitazone may pose a significant risk of adverse events. Clinical trial findings on the use of metformin in AD are limited and controversial, taking into consideration the possibility that vitamin B12 deficiency, often observed in metformin use, may increase cognitive impairment and AD risk. Metformin should also be considered in selected patients with prediabetes according to the American Diabetic Association criteria [96]. Concerning the role of incretins and incretin analogues in the brain, it can be safely assumed that it is in many ways neuroprotective. Although data from animal experiments with incretins were very promising, research in humans has shown contradictory results. Therefore, the role of incretins in AD treatment in humans needs to be further investigated. Taking into consideration that systemic administration of insulin is associated with an increased risk of hypoglycemia, the therapeutic use of insulin has begun to be tested both in clinical and preclinical studies. Given its beneficial impact and the absence of serious side effects, insulin is considered a promising therapeutic agent for AD treatment.

Further consideration is needed in the design of AD treatments including improvements in patient selection, identification of a wider range of biomarkers that adhere to the multifactorial nature of AD, and the study of genetic factors for better understanding of genotype–environment interaction. APOE4 is the strongest genetic risk factor for AD and is an important modulator of the intranasal insulin effects. Further understanding of the vital role that the APOE4 genotype plays on insulin resistance and regulation will eventually lead to developing more individualized treatment strategies for AD patients. It should be noted that glycemic variability and prediabetes may also be involved in other neurodegenerative diseases with neuropathological findings similar to AD, such as Progressive Supranuclear Palsy and Corticobasal syndrome [97].

### 3.6. Limitations

This review study has some limitations. Due to the heterogeneity in the results of the studies presented, it is difficult to come to robust conclusions about the role of antidiabetic drugs in AD treatment. The design, sample size and outcome measures varied between studies. Moreover, some studies had small sample size and short treatment duration.

## 4. Conclusions

AD and T2DM are two of the most pressing epidemics of recent years [98]. It seems that antidiabetic agents may improve cognition as well as modify disease biomarkers in MCI and AD patients. Intranasal insulin shows great promise for AD treatment and its beneficial role is modulated by ApoE genotype status [99]. Despite the encouraging results, there is not yet sufficient evidence to support the use of antidiabetic drugs for AD treatment, and further studies are needed in order to confirm their therapeutic potential.

## Figures and Tables

**Table 1:** Antidiabetic drugs for AD treatment in humans.

Study/Year	Treatment	Study Population	Outcomes
1. Reger et al., 2008 [25]	Intranasal insulin	MCI	Improvements in working memory and cognition.
2. Reger et al., 2006 [26]	Intranasal insulin	AD	Improvements in cognition for APOE4 negative patients.
3. Craft et al., 2012 [39]	Intranasal insulin	AD	Improvements in cognitive and functional ability.
4. Claxton et al., 2015 [24]	Intranasal insulin	AD and MCI	Improvements in cognitive, verbal, and audiovisual memory.
5. Ng et al., 2014 [40]	Metformin	T2DM	Reduction in the risk of cognitive impairment.
6. Hsu et al., 2011 [41]	Metformin	T2DM	Reduction in the risk of dementia by 24%.
7. Koenig et al., 2017 [42]	Metformin	MCI	Positive effect on executive function, as well as some improvements in memory and attention.
8. Luchsinger et al., 2016 [43]	Metformin	MCI	Significant improvement in verbal memory.
9. Moore et al., 2013 [44]	Metformin	AD	Increased risk of cognitive impairment.
10. Imfeld et al., 2012 [45]	Metformin	T2DM	Increased risk of cognitive impairment.
11. Gejl et al., 2016 [46]	Liraglutide	AD	Moderate neuroprotective effects expressed withimprovements in cerebral glucose metabolism.
12. Gold et al., 2010 [47]	Rosiglitazone	AD	No benefit was observed with administration.
13. Watson et al., 2005 [48]	Rosiglitazone	AD and MCI	Improvements in attention and delayed recall.
14. Risner et al., 2006 [49]	Rosiglitazone	AD patients non- APOE4 carriers	Improvements in ADAS-Cog.
15. Abbatecola et al., 2010 [50]	Rosiglitazone	T2DM	Protection against cognitive impairment.
16. Hanyu et al., 2009 [51]	Pioglitazone	AD and DM	Cognitive and metabolic improvements.
17. Sato et al., 2011 [52]	Pioglitazone	AD and T2DM	Improvements in cognitive ability and cerebral blood flow to the parietal lobe.

## Data Availability

Not applicable.
